# How effective is dentin autograft for socket preservation and implant site preparation: A systematic review protocol.

**DOI:** 10.12688/f1000research.144522.1

**Published:** 2024-03-21

**Authors:** Karthik Sivaraman, Eti Rajwar, Aditi Chopra, George Cherukara, Shubhankar Mehrotra, Namrata Datta, Bindhu Koshy

**Affiliations:** 1 Department of Prosthodontics, Manipal College of Dental Sciences, Manipal, Manipal Academy of Higher Education, Manipal, Karnataka, 576104, India; 2 Public Health Evidence South Asia, Department of Health Information, Prasanna School of Public Health, Manipal Academy of Higher Education, Manipal, Karnataka, 576104, India; 3 The George Institute for Global Health India, New Delhi, Delhi, 110025, India; 4 Department of Periodontology, Manipal College of Dental Sciences, Manipal, Manipal Academy of Higher Education, Manipal, Karnataka, 576104, India; 5 School of Dentistry, University of Dundee, Dundee, Scotland, UK; 6 Specialist Referral Practice, Regents Street, BUPA Dental Care, Bristol, UK

**Keywords:** Dentin, Bone, Bone graft, Implants, Tooth Extraction, Socket preservation, Regeneration, Healing, Autograft, Periodontal surgery

## Abstract

**Background:**

Socket preservation is a surgical procedure aimed at preserving the dimensions of the alveolar bone following tooth extraction. It is performed by filling the extraction socket with bone graft material with or without a barrier membrane. Recently, dentine obtained from extracted teeth has been tried as an autograft for socket preservation. Studies have compared the use of dentin to other bone grafts, however, systematic reviews evaluating the efficacy of dentin for socket preservation are limited. Hence, this systematic review protocol is proposed to generate evidence on the efficacy of dentin as a viable alternative to other bone graft materials for socket preservation.

**Methods:**

This systematic review protocol was prepared according to the Methodological Expectations of the Cochrane Intervention Reviews (MECIR) guidelines. It will be conducted using the Cochrane Handbook for Systematic Review of Interventions. PubMed, Scopus, Web of Science, EMBASE, Epistemonikos, Cochrane Central, and EBSCO databases and clinical trial registries, will be searched for all randomized controlled trials (RCTs) and non-randomized studies that have used autologous dentin graft (either in particulate/putty, or/matrix form) for socket preservation. The radiographic and clinical assessment of bone and soft tissue healing of the preserved sockets along with patient-related outcomes following surgery will be assessed. The risk of bias assessment of the RCTs and Non-RCTs will be assessed using the ‘Cochrane Risk of Bias assessment tool (ROB II) and ROBINS-I respectively. The certainty of evidence will be assessed by the GRADE approach.

**Discussion:**

This evidence is important for dental clinicians and the public to make an informed decision when choosing graft material for socket preservation. The extracted teeth are considered biological waste; however, this evidence provides scope for using a less invasive autograft for bone regenerative procedures.

**Systematic review registration:**

PROSPERO: CRD42021201958 (Registered on 15/02/2021).

**List of abbreviations**
BMP: Bone morphogenic protein
CI: Confidence interval
DDM: Demineralized dentin matrix
ECM: Extracellular matrix proteins
GF: Growth factors
GRADE: Grading of Recommendations, Assessment, Development, and Evaluation
NRS: Numeric Rating Scale
OR: Odds ratio
POWF: Pulsed oscillation waveform
PRISMA P: Preferred Reporting Items for Systematic Review and Meta-analysis Protocols
RCT: Randomized controlled trial
RFA: Radiofrequency analysis
RR: Risk ratio
TGF-β: Transforming growth factor
VAS: Visual Analog Scale

## Introduction

Dental implants are increasingly being used as a promising modality for replacing missing teeth with superior biological, functional, and aesthetic properties. ^1^ However, one of the important requirements for placing implants is the presence of favorable volume and architecture of the available bone at the time of placement. Many times, adequate bone height and width may not be present to place an implant owing to resorption of the alveolar ridge following extraction. ^2^^–^^5^ Approximately 29 to 63% of horizontal bone loss and 11 to 22% of vertical bone loss occur within the first six months following tooth extraction. ^6^ The crest of the alveolar ridge also shifts lingually with more bone loss occurring in the buccolingual dimension (4.5 to 6.1 mm) compared to mesiodistal dimensions. ^7^^–^^14^ This reduction in bone volume and thinning of the buccal cortical plates affects the overlying soft tissues, and in turn affects the esthetic, phonetic, and functional outcomes after prosthetic rehabilitation. ^10^^–^^14^ Studies have noted that a loss of 2.46 to 4.56 mm ridge width occurs at sites without socket preservation compared to a reduction of 1.14 to 2.5 mm in preserved socket sites. ^15^^–^^25^ Additionally, preserving sockets immediately following extraction reduces the need for additional ridge augmentation procedures at the time of implant placement by five times when compared to unassisted socket healing. ^26^^–^^28^ Studies have also noted that approximately 20.8 % of non-preserved sockets need additional grafting for implant placement compared to only 9.9 % of preserved sockets. ^28^^–^^35^ Thus, to prevent the inevitable resorption of the alveolar ridge, reduce the physiologic bone loss, and retain the volume and quality of bone and associated soft tissue profile, sockets are preserved with bone grafts with and without barrier membranes. ^15^^–^^24^

Sockets can be preserved with either an autograft, xenograft, allograft, or alloplasts. Among all these bone grafts, autografts have the highest bone regenerative potential. ^20^^–^^22^ Autografts can be harvested from edentulous ridges, extraction sockets, mandibular ramus, symphysis, maxillary tuberosity, tibia, iliac crest, and calvarium. ^20^ However, procuring autograft is difficult and requires superior clinical skills. Additionally, many patient-related complications, such as post-operative infection, root resorption, ankylosis, and increased patient morbidity have been noted with the use of autografts. ^20^^–^^24^ To overcome these limitations novel bone graft materials with osteogenic potential are being researched.

Recently, whole tooth, enamel, and dentin have been tried as autograft material for various surgical procedures. ^23^^–^^42^ Among these dentin autografts are being used owing to their high osteoinductive potential. The composition, physical, and chemical properties of dentin are also similar to that of alveolar bone. ^30^ Dentin also contains insulin-like growth factor, bone morphogenetic protein (BMP), and transforming growth factor-beta (TGF-β) which promotes bone regeneration. ^25^^–^^29^ It is available in three forms: demineralized dentin matrix, partially demineralized dentin matrix, and un-demineralized dentin. Dentin bone graft is used for various periodontal and oral surgical procedures such as sinus lift, periodontal defects, ridge augmentation, socket preservation for implant placement, and the treatment of peri-implantitis. ^26^^–^^42^ However, there are still various uncertainties and limited systematic reviews related to its use for socket preservation compared to other bone graft materials. The PubMed search with the keyword dentin bone graft yielded only six systematic reviews, with none comparing dentin to other bone graft materials for socket preservation. There is limited data that quantitatively substantiates whether sockets preserved with dentin bone graft are better than those preserved with other bone graft material. Additionally, the understanding of the effect of dentin bone graft on the nature of bone healing and soft tissue healing around the preserved sockets, stability of implants placed in the sockets preserved with dentin, and postoperative complications after using dentin are limited. Thus, this systematic review aims to synthesize evidence on the effectiveness and efficacy of dentin autograft as a graft material for socket preservation compared to allograft, alloplast, and xenograft materials. This evidence is crucial for clinicians, patients, and other stakeholder groups in making evidence-based choices regarding the use of dentin as a bone graft material.

## Objectives

### Primary objective

To assess the clinical and radiographic changes in the height and width (in mm) of the extraction sockets preserved with dentin autograft (particulate/putty) compared to allograft, alloplast, and xenograft materials.

### Secondary objectives

1. To evaluate and compare the clinical and radiographic change in the quality of bone and soft tissue healing in sockets preserved with dentin bone graft compared to sockets preserved with other bone grafts (allograft, alloplast, or xenograft).
2. To assess and compare the details of the type of dentin bone graft used, method of application, duration of surgical procedure, patients’ compliance, time of surgery with dentin bone graft compared to sockets preserved with other bone graft (allograft, alloplast or xenograft).
3. To assess and report prognosis and any adverse events/effects or postoperative complications associated with the use of dentin autografts for sockets preservation.

## Methods

The proposed systematic review will be conducted according to the Cochrane Handbook for the Systematic Review of Interventions, ^43^ Methodological Expectations of the Cochrane Intervention Reviews (MECIR) guidelines ^44^ and the Preferred Reporting Items for Systematic Review and Meta-analysis Protocols (PRISMA-P) checklist. ^45^^,^^48^ The systematic review protocol has been registered in the International Prospective Register of Systematic Review PROSPERO with registration number - CRD42021201958 on 15/02/2021. Differences between the protocol and the final review, if any, because of the methodological alterations due to the unavailability of data, will be reported in the final review.

### Criteria for considering studies for the proposed systematic review

The eligibility criteria are described using the Participant-Intervention-Comparison-Outcome-Study Design (PICOS) framework, which also indicates the key components of the research question.

*Types of participants*

For the proposed systematic review, studies with healthy participants who are above 18 years of age and have undergone a socket preservation procedure following tooth extraction will be included.

Studies with participants who have or have not received any dental implants, either at the time of socket preservation or following socket preservation will also be included. Participants who have undergone tooth extraction because of any of the following reasons will be included: unrestorable tooth due to caries or fracture; avulsion of a tooth following trauma; root resorption (internal or external); tooth fracture that cannot be restored; endodontic infection or periodontal disease that cannot be addressed with endodontic and periodontal therapy; failure of root canal treatment, a tooth with grade III mobility and more than 50% bone loss, Grade IV furcation defect (based on Glickman classification) with through and through bone defect that cannot be regenerated and needs an extraction.

Following exclusion criteria will be followed:

1. Studies with participants who have a medical history such as diabetes, hypertension, cardiovascular, renal diseases, respiratory, neurological disorders, cancer, psychiatric conditions, autoimmune disease, or pregnancy.
2. Studies with participants suffering from conditions or diseases that might impair bone healing or metabolism, for example, osteoporosis, vitamin D deficiency, and use of anti-resorptive therapy such as bisphosphonates.
3. Studies with participants who use tobacco products, areca nut, gutka, or supari, as smoking. The above factors may influence the uptake of the graft material and or dental implants and hence have been used as exclusion criteria.
4. Studies with participants undergoing chemotherapy or radiation therapy as these treatment modalities compromise angiogenesis and impair bone healing.

*Types of interventions*

Studies, where the extraction sockets are preserved with any form of dentin bone graft (demineralized, partially demineralized, or not demineralized) in any form (particulate/putty/matrix/gel), size, or application method with or without a barrier membrane will be included. Studies, where the extraction sockets are preserved with dentin (any particle size) and compared to other bone grafts such as other autografts, alloplasts, allografts, and xenografts with a minimum follow-up of three months will be included. Since bone healing requires a minimum of three months, studies providing a three-month follow-up will be included. Studies where implants are placed immediately following socket preservation will also be included. Studies with both infected and non-infected sockets will be included. Studies where the whole tooth was used as a graft material, will not be considered.

*Types of comparators*

Studies, where dentin bone graft is compared with any of the following bone graft material, will be included: autografts (enamel; autologous bone graft from any anatomic site such as chin, ramus, maxillary tuberosity; fresh socket, bone chips, osseous coagulum, bone blend); allograft (Demineralized freeze-dried bone graft and freeze-dried bone graft); xenograft (bovine, porcine, coral-based xenograft); and alloplast (hydroxyapatite; plaster of Paris; tricalcium phosphate; calcium phosphosilicate; calcium phosphate; calcium silicate, *etc.*).

*Types of outcomes*

The following outcomes will be considered in the proposed review: 1) Clinical and radiographic changes in the height and width (in mm) of the socket before and after socket preservation with dentin bone graft compared to other bone graft; 2) Changes in quality of bone following socket preservation; 3) clinical assessment of soft tissue (in terms of gain in the width of keratinized gingiva) following socket preservation; 4) details of the type of dentin bone graft used, method of application, duration of surgical procedure 5) occurrence of any immediate and delayed post-operative complications following socket preservation surgery.

*Types of studies*

In the proposed systematic review, randomized controlled trials (RCTs), the gold standard study design for assessing the effectiveness of an intervention will be used as the preferred study design for inclusion. RCTs with parallel-group, individual participant, or cluster-allocation designs comparing the independent effects of the intervention on the comparison group will all be included. Non-randomized trials such as pre-post (controlled before-after) and quasi-experimental studies will also be included. Case series with more than 10 subjects and a follow of at least three months would be included. Animal studies, case series with fewer than 10 subjects, letters to the editor, and case reports will all be excluded from inclusion. Only manuscripts published in the English language will be included in the proposed systematic review.

*Context or setting*

Studies conducted in hospitals, dental clinics, university hospitals, and other relevant healthcare facilities, dependent on the country where the study was conducted, will be included in the review. Studies, where treatment was provided by specialists and non-specialists, will be included in the review.

### Search strategy and information sources

A search strategy was developed using keywords identified from the preliminary search and following agreement between the research team (KS, AC, ER) and a search expert (GC, BK). It included Medical Subject Headings (Mesh) terms, free text terms, and Boolean (AND, OR), as well as the components of the PICO framework. The advanced search strategy for PubMed, one of the databases to be searched is given in Table 1. An electronic search for English language publications in PubMed, Scopus, Web of Science, EMBASE, Epistemonikos, EBSCO (Dentistry and Open Access), and Cochrane CENTRAL databases will be conducted. Additionally, ProQuest, World Clinical Trial Registry, and Clinical Trials Registry of India (CTRI) will be searched for ongoing clinical trials. The corresponding authors for the ongoing clinical trials will be requested to share the results (if available). In addition, the bibliography section of the following journals will be searched for any articles in press: Journal of Periodontology, Journal of Dental Research, Journal of Periodontal Research, Journal of Clinical Periodontology, Journal of Clinical Oral Implant Research, Journal of Implantology, Journal of Clinical Implants and Related Research. We will also check references of the included studies for other relevant articles. Preprint servers such as BioRxiv, Research Square, and MedRxiv will be screened for relevant articles on dentin as bone graft material used in socket preservation. Doctoral theses, conference proceedings, and industry studies published in company publications will also be screened for relevant literature.

**Table 1.** Search strategy (PubMed).

(“dental implants”[MeSH Terms] OR “Autogenous tooth bone graft”[Title/Abstract] OR “Autogenous demineralized dentin graft”[Title/Abstract] OR “Tooth derived bone graft”[Title/Abstract] OR “dentin”[MeSH Terms] OR “dentin”[Title/Abstract] OR “Autogenous dentin graft”[Title/Abstract] OR “Particulate dentin”[Title/Abstract]) AND (“transplantation, autologous”[MeSH Terms] OR “autografts”[MeSH Terms] OR “allografts”[MeSH Terms] OR “Alveolar ridge augmentation”[MeSH Terms] OR “autograft*”[Title/Abstract] OR “allograft*”[Title/Abstract] OR “heterograft*”[Title/Abstract] OR “xenograft*”[Title/Abstract] OR “autologous graft*”[Title/Abstract] OR “allogenic bone graft*”[Title/Abstract] OR “autogenous graft*”[Title/Abstract] OR “bone graft*”[Title/Abstract] OR “autogenous bone graft*”[Title/Abstract] OR “Alveolar ridge augmentation”[Title/Abstract]) AND (“peri-implantitis”[MeSH Terms] OR “paresthesia”[MeSH Terms] OR “pain, postoperative”[MeSH Terms] OR “guided tissue regeneration”[MeSH Terms] OR “peri-implantitis”[Title/Abstract] OR “periimplantitis”[Title/Abstract] OR “post-operative pain*”[Title/Abstract] OR “postoperative pain*”[Title/Abstract] OR “guided tissue regeneration”[Title/Abstract] OR “bone regeneration”[Title/Abstract] OR “peri implant infection*”[Title/Abstract] OR “peri implant infection*”[Title/Abstract] OR “paresthesia*”[Title/Abstract] OR “Dysesthesia”[Title/Abstract] OR “socket-length”[Title/Abstract] OR “socket-length”[Title/Abstract] OR “Socket-height”[Title/Abstract] OR “Socket preservation”[Title/Abstract] OR “Implant mobility”[Title/Abstract] OR “post-operative complication*”[Title/Abstract] OR “post-operative complication*”[Title/Abstract] OR “post-operative adverse effects”[Title/Abstract] OR “post-operative adverse effect*”[Title/Abstract] OR “post-operative healing”[Title/Abstract] OR “post-operative healing”[Title/Abstract] OR “post-operative wound management”[Title/Abstract] OR “post-operative wound management”[Title/Abstract] OR “Post-surgical pain”[Title/Abstract] OR “Postsurgical pain”[Title/Abstract] OR “implant survival”[Title/Abstract] OR “implant stability”[Title/Abstract] OR “implant prognosis”[Title/Abstract]) AND (“humans”[MeSH Terms] AND “english”[Language]) AND “humans”[MeSH Terms] AND “english”[Language]) AND (“humans”[MeSH Terms] AND “english”[Language])) AND ((humans [Filter]) AND (AND (english [Filter]))

### Study selection (screening process)

The search results will be managed using the Microsoft Office (MS) Excel software. After the removal of duplicates, four reviewers (KS, AC, SM, ND) will independently select studies using the inclusion and exclusion criteria. The screening process will be performed in two stages. In the first stage, the titles, and abstracts of the studies will be independently screened. Studies that do not qualify according to the eligibility criteria and those with inadequate data or information will be excluded at the title/abstract (Ti/Ab) screening stage. In the second stage, full-text screening of the included studies will be performed independently by the four reviewers (KS, AC, SM, ND). They will also perform hand-searching of the journals listed above and screen the potential articles for inclusion. Any disagreements regarding inclusion will be resolved initially by discussion between the reviewers (GC) and if unresolved the other reviewers (BK, KS) will be consulted for agreement; a majority decision will be adopted. A pre-designed screening protocol will be used to select studies ( Table 2).

**Table 2.** Screening protocol.

**1.**	**Title and abstract screening**		
A.	Is the study published in English? AND is it published in the year 2000 or later?	If the answer to both the components is ‘yes’, Go to B	If it is non-English or published before 2000 then exclude the study
B.	Does the study involve one of the following designs or analysis: Randomized controlled trials and non-randomized clinical trials including pre-post analysis and quasi-experimental studies	If the answer is ‘yes’ OR it is not clearly stated in the abstract, Go to C	If the publication is a commentary, perspective, editorial, review, conference abstract, OR policy paper that does not provide details of the implementation of the intervention: exclude the study
C.	Does the study describe the intervention in the form of dentin autografts in comparison to other types of bone grafts for socket preservation about implant treatment?	If the answer is ‘yes’ OR it is not clearly stated in the abstract, Go to D	If the answer is ‘no’ then exclude
**2.**	**Full-text screening**		
D.	Did the study involve the adult population i.e. 18 yrs. and above? And if the study involves healthy patients without any medical history such as diabetes, hypertension, cardiovascular, or renal diseases nor be on any medication for these or other systemic conditions.	If it is ‘yes’, Go to E	If no, or if the study involves participants with any systemic disease that might impair bone metabolism, anti-resorptive therapy such as bisphosphonates, pregnancy, psychiatric conditions, and heavy smokers (>10 cigarettes), including the use of smokeless or chewing form of tobacco, areca nut or supari. If the study includes participants undergoing chemotherapy or radiation therapy, in such cases, exclude the study
E.	Does the study involve one of the following designs or analyses: Randomized controlled trials and non-randomized clinical trials including pre-post analysis and quasi-experimental studies?	If it is ‘yes’, Go to F	If ‘no’, exclude the study
F.	Does the study describe the intervention in the form of dentin autografts in comparison to other types of bone grafts for socket preservation about implant treatment?	If the answer is ‘yes’ Go to G OR If you are in doubt, then flag for discussion	If ‘no’, exclude the study
G.	Did the study measure outcomes of our interest e.g. radiographic and clinical assessment of the bone and soft tissue healing in the preserved socket, type of dentin bone graft and nature of the processing of the graft; post-operative complications, etc.?	If ‘yes’, including the study for data analysis	If ‘no’, exclude the study

### Data extraction

Three reviewers (KS, AC, ND, SM) will perform data extraction independently, using a pre-designed pilot-tested data extraction sheet. Any disagreements during the extraction process will be resolved initially by discussion between three reviewers, and if still unresolved BK and GC will be consulted. A majority decision will be adopted as final. In case of unpublished and missing information/data, the study authors will be contacted to request the required information. If adequate data are available, the review team will perform additional relevant statistical analysis.

### Outcomes assessed

The outcomes considered in the proposed review are:

*Primary outcomes*:

i. Clinical and radiographic assessment of the overall bone volume gain or otherwise, following socket preservation as measured by the gain or otherwise, in the mesiodistal, buccolingual, and apicocoronal dimensions of the socket before and after the intervention (in mm) will be measured. This can be measured by bone-sounding/trans-gingival probing or any radiographic assessment. The changes in the proximal/interdental bone levels at teeth-bounding extraction sites will also be noted.
ii. The nature of bone, and quality of bone as assessed by any clinical or radiographic method will be considered. Radiographic assessment of bone volume or quality as measured by changes in the density of the trabecular pattern (no/mm ^2^); changes in the trabecular pattern; ratio of cortical to cancellous bone in terms of Hounsfield unit (HU) in the extraction socket pre- and post-intervention (mm ^2^); or any other techniques reported in respective studies will be considered. The stability of implants in sockets preserved with dentin bone graft compared to other bone grafts is of interest.

If the clinical method is not used and the radiographic method is used in the studies to provide the change in the dimension of the socket and it does not provide three-dimensional information, the two-dimensional measurement will be recorded. The method of assessment will also be recorded. These outcomes have been chosen as the primary outcomes because the quality and quantity of bone and soft tissue following socket preservation are the most crucial outcomes to define success.

*Secondary outcomes*:

i. The health of soft tissue around the socket preserved, as measured by the health of the gingival tissues and gain in the amount of keratinized tissue after socket preservation at baseline and post-treatment, as measured by any histomorphometric analysis or manually by any periodontal probe will be noted.
ii. Percentage/amount of bone graft remaining in the socket post-intervention (at each recall) reported by surgical re-entry, histology, or radiographic imaging technique will be considered.
iii. Presence or absence of any postoperative complications at the time of surgery, immediately after surgery; within 24 hours, or delayed after socket preservation will be considered. Presence or absence of non-healed sockets, delayed socket healing, exposure of barrier membrane, loss of graft material, presence and presence of soft tissue defects on the alveolar ridge; presence or absence of any suppuration, pus discharge, excessive bleeding, dehiscence and opening of the wound, fenestration on the buccal or lingual aspect of the closed wound, recession of soft tissue following surgery, postoperative pain, persistent pain or dysesthesia following treatment as measured by any pain scale such as Visual Analog Scale (VAS), Numeric Rating Scale (NRS), and The Wong-Baker face pain scale following socket preservation are other will be considered.
iv. Time required between socket preservation and implant placement; nature of osseointegration if implants were placed at the time of socket preservation will be considered.
v. Health of the tooth used for procurement of dentin; type of dentine used, method of processing of dentin; time of utilization of tooth following extraction; method of storage of dentin, mode of application of dentin (with or without any device) and particle size of dentin graft (if mentioned); use of dentin with or without a barrier membrane will be considered.

### Risk of bias (ROB) assessment

The risk of bias will be assessed using the revised Cochrane Risk of Bias Assessment Tool (ROB2). ^46^^,^^47^ Two review authors (KS, AC) will independently assess the risk of bias in the included studies. Any disagreements will be resolved via discussion, if consensus cannot be reached, then a majority decision will be adopted after consulting the other two reviewers (BK, GC). The following domains will be assessed for risk of bias: 1. Randomization process; 2. Bias resulting from deviations from intended interventions; 3. Bias resulting from missing outcome data; 4. Bias resulting from measurement of the outcome; 5. Bias resulting from selection of the reported result; 6. Bias in the identification or recruitment of individual participants within clusters. If cluster-RCTs are included, whether the reported data analysis had appropriately taken account of the aggregate nature of the data will be determined. The identified risks shall be categorized as low risk, high risk, and risk with some concern. The ‘effects of assignment’ will be considered as the effect of principal interest to assess the risk of bias.

Quality assessment of non-randomized studies will be performed using the Risk of Bias In Non-Randomized Studies of Interventions (ROBINS-I). ^47^ As per this tool, bias in a non-randomized trial will be assessed for the following seven domains: 1) Bias due to confounding, 2) Bias in the selection of participants into the study, 3) Bias in the classification of interventions, 4) Bias due to deviations from intended interventions, 5) Bias due to missing data, 6) Bias in measurement of outcomes, 7) Bias in selection of the reported result. The assessed risk will be categorized as low risk, moderate risk, serious risk, and critical risk.

### Measures of effects

In the case of continuous data, Mean Difference (MD) and Standard Mean Difference (SMD) with 95% Confidence Interval (CI) will be used to estimate the effects of the intervention. Odds Ratio (OR) and Risk Ratio (RR) with 95% CI will be used for categorical data.

### Unit of analysis

The participant will be the unit of analysis for individual randomized trials, however, a study cluster will be considered as the unit of analysis for cluster-randomized trials. The impact of data clustering will be considered and the unit of analysis will be adjusted accordingly during analysis.

### Data synthesis

Meta-analysis will be performed in case homogenous data is obtained from the included studies. I ^2^ statistic will be used to assess statistical heterogeneity, describing the percentage of the variability in effect estimates that is due to heterogeneity rather than chance. Based on the I ^2^ statistic, the level of heterogeneity will be categorized as follows:

• 0% to 40%: might not be important
• 30% to 60%: may represent moderate heterogeneity
• 50% to 90%: may represent substantial heterogeneity
• 75% to 100%: considerable heterogeneity

A fixed-effects model will be used for meta-analysis. A forest plot will be used to report the results of the meta-analysis. A narrative synthesis, with tables and figures for explanation, will be performed when meta-analysis is not possible in case of more than 50% ‘clinical,’ ‘methodological,’ and ‘statistical’ heterogeneity.

### Sub-group analysis

If possible, sub-group analysis will be performed based on the height and width of the socket before and after socket preservation; quality of bone formed before and after socket preservation in dentin compared to other bone grafts; nature of soft tissue before and after socket preservation in dentin compared to other bone graft; post-operative complications following socket preservation.

### Assessment of reporting bias

Funnel plots will be used for the identification of reporting bias. The identification of reporting bias will be done based on the visual assessment of the funnel plot, where a symmetrical funnel plot will be interpreted as the absence of reporting bias and an asymmetrical plot will indicate the presence of reporting bias.

### Sensitivity analysis

If sufficient data is available, a sensitivity analysis will be performed to assess the robustness of the results. This will be done using studies with a ‘low risk of bias’.

### Assessment of certainty of evidence

The certainty of evidence will be assessed using the Grading of Recommendations, Assessment, Development, and Evaluation (GRADE) approach. This approach grades evidence for five domains 1) risk of bias, 2) indirectness, 3) inconsistency, 4) imprecision, and 5) publication bias. The level of certainty of evidence will be classified as high, moderate, low, and very low, using the GRADE Pro GDT software, independently by three of the review authors (KS, AC, and ER). Disagreements will be resolved by discussion and in case of non-consensus, the two other authors (BK and GC) will be consulted, and a majority decision taken. The certainty of evidence will be reported for the following seven outcomes 1) Changes in the dimensions (height and width) of the alveolar ridge following grafting; 2) Nature of bone and soft tissue healing following socket preservation; 3) Postoperative complications (presence and absence of pain, dysesthesia, suppuration, marginal bone loss or loss of graft material.

### Study status

The review is currently in the full-text screening stage.

## Discussion

The proposed systematic review will assess the quality of the evidence base available regarding the efficacy and efficiency of using dentine bone graft material in socket preservation. The review will help to understand whether dentin has a superior osteogenic potential than other bone grafts and what future complications one should note when using dentin. This will help to understand if dentin is a good alternative to conventional autografts like ramus graft and chin graft which involve many patient-related complications and morbidity. ^18^^–^^20^ Since many times tooth is discarded as a biological waste, the utilization of the same tooth for regeneration of bone defect would preclude the need for additional bone graft material. This would help patients to obtain a cheaper, affordable, and relatively easier option compared to other autografts. Hence the evidence is crucial for the patients and clinicians who wish to make informed decisions about choosing dentin autograft, in resource-constrained settings. ^23^^–^^32^ We have involved relevant stakeholders such as clinicians, patients, and researchers for the identification of research uncertainties and the formulation of the research question. After consultation with the stakeholders, the need for a non-invasive, cheaper, and more comfortable option for procuring autografts was deemed necessary. We also took feedback from patients in our clinics regarding their experience following surgery where autograft was procured from the chin and ramus. There is a need to explore this research knowledge and explore the efficacy of dentin as bone graft material for socket preservation and at the same it is important to understand the clinical outcomes and patient-related outcomes after surgery using dentin as a bone graft material. The review will also cue the dentist to understand the probable complications associated with dentin bone graft. Thus, the comprehensive evaluation of research and the resulting consolidated up-to-date evidence will help clinicians make evidence-informed decisions while choosing graft material. Additionally, the review will also propose further studies required to address gaps identified in the knowledge created so far on dentine as a graft material. It is hoped that collaborative research with clinicians, scientists, and the industry will be promoted as a result of the planned review. However, one should note that one of the limitations of the proposed review is that the evidence will be based on English language literature only. Although this review aims to explore, the role of dentin as graft material for socket preservation, we would not be able to provide strong evidence on the stability, prognosis, and long-term survival of the implants as these factors have many confounding variables that cannot be addressed and is not within the scope of this review. However, future studies should explore the prognosis and survival rate of implants in sockets preserved in the dentin compared to other bone grafts.

## Data Availability

No data are associated with this article. Figshare: PRISMA-P checklist for ‘How effective is dentin autograft for socket preservation and implant site preparation: A systematic review protocol.’ https://doi.org/10.6084/m9.figshare.25195910. ^48^ Data are available under the terms of the Creative Commons Attribution 4.0 International license (CC-BY 4.0).
