# Supplementary material for: How effective is dentin autograft for socket preservation and implant site preparation: A systematic review protocol
Source: F1000Res. 2024 Aug 1;13:204. Originally published 2024 Mar 21. [Version 2] doi: 10.12688/f1000research.144522.2 (PMC11263907; doi:10.12688/f1000research.144522.2)
Supplement: Peer review history

# Reviewer report: Reviewer response for version 2

**DOI:** 10.5256/f1000research.169766.r309907

**Published:** 2024-08-07

**Authors:** Lydia N. Melek

**Affiliations:** 1 Alexandria University, Alexandria, Egypt

Comments have been adequately addressed by the authors.

Is the study design appropriate for the research question?

Yes

Is the rationale for, and objectives of, the study clearly described?

Yes

Are sufficient details of the methods provided to allow replication by others?

Yes

Are the datasets clearly presented in a useable and accessible format?

Not applicable

Reviewer Expertise:

Oral and Maxillofacial surgery with a special interest in dental implantology and grafts. I have several publications on the use of dentin graft.

I confirm that I have read this submission and believe that I have an appropriate level of expertise to confirm that it is of an acceptable scientific standard.

# Reviewer report: Reviewer response for version 1

**DOI:** 10.5256/f1000research.158329.r295742

**Published:** 2024-07-22

**Authors:** Marwa Madi

**Affiliations:** 1 Imam Abdulrahman Bin Faisal University, Dammam, Saudi Arabia

The study is well written and the methodology is explained in a detailed and organized manner. Since the objective of the review is clinical and radiographic findings as well as soft tissue healing following socket preservation using dentin allograft I think the authors need to exclude all animal studies and include only clinical studies.

**Strength of the proposal:**

- **The proposal addresses a novel approach by investigating the use of dentin autografts for socket preservation, which is an emerging area in dental research. This originality is relevant and significant for students at this level. The proposal is grounded in a well-established scientific premise, highlighting the importance of alveolar ridge preservation and the potential benefits of using dentin as a graft material. The background information provided is comprehensive and supports the rationale for the study.**
- **The methodology is robust, following the Cochrane Handbook for Systematic Reviews and adhering to PRISMA-P guidelines. The use of multiple databases for literature search, clear inclusion and exclusion criteria, and detailed data extraction and analysis plan demonstrate feasibility and rigor.**

**The weakness of the proposal: Focus** **on Specific Population**:

- The study focuses on healthy adults, potentially overlooking how dentin autografts might perform in populations with comorbidities or different age groups.

**Limited Scope of Comparative Studies**: While the proposal compares dentin autografts with other types of grafts, it does not account for potential variations in outcomes based on patient demographics or specific clinical conditions.

Is the study design appropriate for the research question?

Yes

Is the rationale for, and objectives of, the study clearly described?

Yes

Are sufficient details of the methods provided to allow replication by others?

Yes

Are the datasets clearly presented in a useable and accessible format?

Yes

Reviewer Expertise:

Periodontology; implant dentistry

I confirm that I have read this submission and believe that I have an appropriate level of expertise to confirm that it is of an acceptable scientific standard.

# Response

**Published:** 2024-07-29

**Authors:** Aditi Chopra

**Affiliations:** Manipal College of Dental Sciences, Manipal, India

Reviewer 2

 Comment 1: Since the review's objective is clinical and radiographic findings and soft tissue healing following socket preservation using dentin allograft, I think the authors need to exclude all animal studies and include only clinical studies.

 Response: We agree with the reviewer, We have added these points to the eligibility criteria.

 Comment 2: The study focuses on healthy adults, potentially overlooking how dentin autografts might perform in populations with comorbidities or different age groups.

 Response: We thank the reviewer for their valuable insight. We agree that the effect of dentin should be checked in diverse populations to establish its validity and efficacy. Hence, we would like to highlight that we would not exclude studies where dentin was used in populations with co-morbidities. This would even help us to do a subgroup analysis and check. We have added these points to our eligibility criteria.

 Comment 3: While the proposal compares dentin autografts with other types of grafts, it does not account for potential variations in outcomes based on patient demographics or specific clinical conditions.

 Response: We agree with the reviewer for this valuable suggestion. Based on the articles we retrieved, we can analyze the clinical outcomes based on variation in variations in demographic and clinical conditions.

# Reviewer report: Reviewer response for version 1

**DOI:** 10.5256/f1000research.158329.r295738

**Published:** 2024-07-08

**Authors:** Lydia N. Melek

**Affiliations:** 1 Alexandria University, Alexandria, Egypt

The systematic review protocol is very well-designed with sufficient feedback about the topic and adequately addresses the current knowledge gap in utilizing the dentin graft as an autograft alternative. The study aim is clear and the methods are reproducible. 

 The authors are advised to add a separate subtitle with the research question of the systematic review stated clearly and concisely for the reader.

 Also, the authors are advised to add the time frame for the studies included in the review. It's mentioned in Table 2 "year 2000 or after" but it's better to add it in the manuscript too.

Is the study design appropriate for the research question?

Yes

Is the rationale for, and objectives of, the study clearly described?

Yes

Are sufficient details of the methods provided to allow replication by others?

Yes

Are the datasets clearly presented in a useable and accessible format?

Not applicable

Reviewer Expertise:

Oral and Maxillofacial surgery with a special interest in dental implantology and grafts. I have several publications on the use of dentin graft.

I confirm that I have read this submission and believe that I have an appropriate level of expertise to confirm that it is of an acceptable scientific standard.

# Response

**Published:** 2024-07-29

**Authors:** Aditi Chopra

**Affiliations:** Manipal College of Dental Sciences, Manipal, India

Firstly I would like to thank the editor and all the reviewers for their valuable suggestions and insightful comments. It has helped us in improving our work. We have tried our best to revise the manuscript as per the comments. The changes are highlighted in yellow.

 Reviewer 1

 Comment 1: The systematic review protocol is very well-designed with sufficient feedback about the topic and adequately addresses the current knowledge gap in utilizing the dentin graft as an autograft alternative. The study aim is clear and the methods are reproducible. The authors are advised to add a separate subtitle with the research question of the systematic review stated clearly and concisely for the reader.

 Response: We have now added a separate subtitle as the focus question as suggested.

 Comment 2: Also, the authors are advised to add the time frame for the studies included in the review. It's mentioned in Table 2 "year 2000 or after" but it's better to add it in the manuscript too.

 Response: We have now added the study duration in the text.
